# Development and magnetic field measurement of a 0.5-m-long superconducting undulator at IHEP

**DOI:** 10.1107/S1600577522006166

**Published:** 2022-06-23

**Authors:** Junhao Wei, Yuhui Li, Xiangchen Yang, Zilin Chen, Xiangzhen Zhang, Xiaojuan Bian

**Affiliations:** a China Spallation Neutron Source, Institute of High Energy Physics, Chinese Academy of Sciences, 1 Zhongziyuan Road, Dongguan, Guangdong 523000, People’s Republic of China; b Institute of High Energy Physics, Chinese Academy of Sciences, 19B Yuquan Road, Beijing 100049, People’s Republic of China

**Keywords:** superconducting undulator, vertical test, magnetic field measurement

## Abstract

Undulators are important devices for accelerator-based light sources whose magnetic field quality determines the photon beam performance. Superconducting-type undulators have been developing rapidly in recent years around the world. The insertion device group at the Institute of High Energy Physics (IHEP) in China started an R&D project to develop prototypes of superconducting undulators (SCUs). A half-meter-long planar SCU has been produced recently. The SCU was designed based on the simulation program *OPERA-3D* giving a period length of 15 mm and a gap of 7 mm. This prototype was manufactured and fabricated precisely, and was then tested by vertically submerging in liquid helium in a Dewar. After quench training several times, the maximum current in the main coils reached 480 A. The magnetic field was measured by Hall probes mounted on a sledge in the middle of the undulator gap. The peak magnetic field reached 1 T. The measurement results indicate that correction coils with suitable current can not only optimize the magnetic first and second integrals but also reduce the phase error, which is expected by design.

## Abstract 2

A 0.5 m-long planar superconducting undulator prototype has been developed at the Institute of High Energy Physics in China. The performance of this prototype was investigated based on a vertical test system.

## 1. Introduction

Undulators, consisting of arrays of dipole magnets, are key devices in advanced light sources for generating high-brilliance photon beams. Compared with permanent undulators, superconducting undulators (SCUs) can achieve higher magnet peak fields at the same period length leading to higher photon brilliance and wider photon energy range (Hwang *et al.*, 2011 ▸; Bahrdt & Ivanyushenkov, 2013 ▸). In recent years, the light source community have been paying more attention to the development of SCUs (Casalbuoni *et al.*, 2018 ▸; Ivanyushenkov *et al.*, 2015 ▸; Xu *et al.*, 2017 ▸; Calvi *et al.*, 2019 ▸).

The insertion device (ID) group at IHEP started an R&D project to produce a 1.5 m-long planar SCU with 15 mm period length. At the current stage, a 0.5 m prototype has been designed and manufactured. The performance of the photon beam emitted from the undulator is strongly dependent on the magnetic field quality. To fulfill the requirement of precise field characterization, a vertical test system was set up to measure the magnetic field distribution of the SCU on-axis. Two superconducting mock-up coils were manufactured and fabricated precisely with 7 mm magnet gap. A brass sledge carried three Hall sensors pulled through the gap along the undulator to measure the local magnetic field. The fabricated SCU coils were hung in a Dewar submerged in liquid helium to ensure the testing temperature at 4.2 K. Three power supplies were used during the tests, one for the main coils and the other two for the correction coils. After several quench training times, the current in the main coils of the SCU reached 480 A while the peak field on-axis exceeded 1 T. The correction coils were also tested at different currents which verified their ability to optimize the first and second integrals of the magnetic field on-axis according to the measured field distribution from the Hall probes. We also observed that the phase of the magnetic field can be improved with a suitable correction current.

In this paper, we introduce the development of our first 0.5-m-long SCU prototype and analyze the magnetic field measured from the Hall probes during the vertical tests. In Section 2, simulation results based on the *OPERA-3D* program are presented. The manufactured SCU prototype is introduced in Section 3. Section 4 describes the vertical test system. The measurement results are given in Section 5, and the paper ends with a conclusion in Section 6.

## 2. Simulation

**Figure 1:**
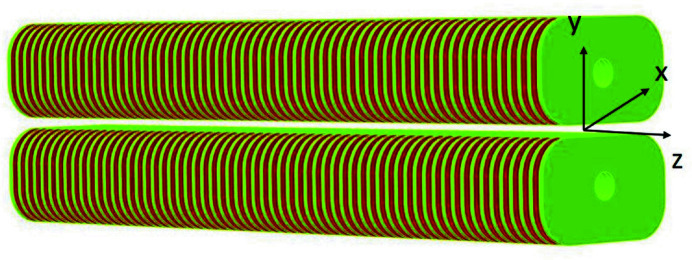
Simulation model with the *OPERA-3D* program.

The SCU structure was designed based on the simulation results using the program *OPERA-3D* (Dassault System, 2021 ▸). Fig. 1 ▸ shows a model of the SCU with 61 coil packs on each iron core. The *x*, *y* and *z* directions are defined in the figure. The period length is 15 mm, and each pole is 3 mm in width. The cross section of the iron core is race-track type with 90 mm width and 47 mm height. The target peak magnetic field on-axis is 1 T for a 7 mm gap. Table 1 ▸ lists some parameters of the SCU model.

**Figure 2:**
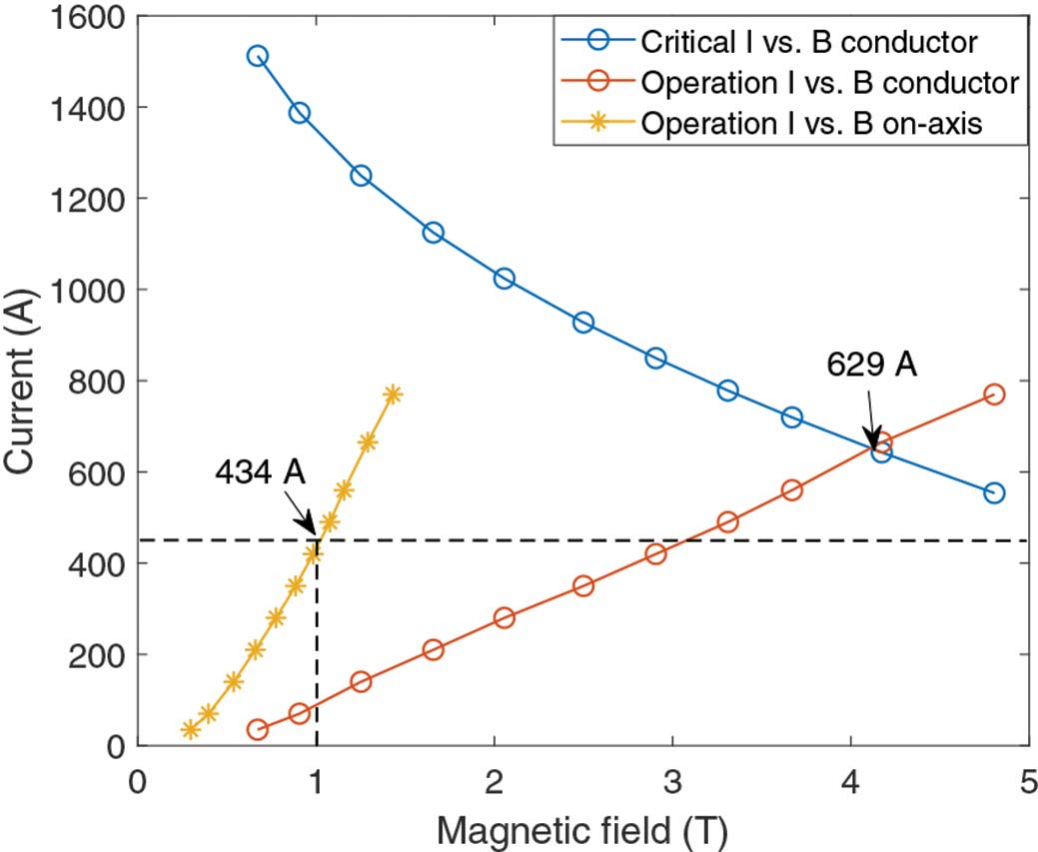
Operation currents (red) and critical currents (blue) with respect to the peak fields in conductors. The yellow points indicate the operation current versus the magnetic field on-axis. The maximum operation current is 629 A and the working current should be 434 A to reach the target peak field of 1 T.

The SCU coils are wound with round NbTi/Cu wires with a diameter of 0.6 mm including 0.1 mm insulation film. The copper to non-copper ratio of the wire is 0.6. The operation current in the NbTi coils must be below the critical current which depends on the maximum magnetic field and the temperature (4.2 K) in the coil packs (Casalbuoni *et al.*, 2014 ▸; Karasev *et al.*, 2013 ▸). Through simulation, we obtained the operation current and the critical current in the NbTi coils versus the maximum magnetic field in the superconductors, and the dependence of the operation current with respect to the magnetic field on-axis, as shown in Fig. 2 ▸.

**Figure 3:**
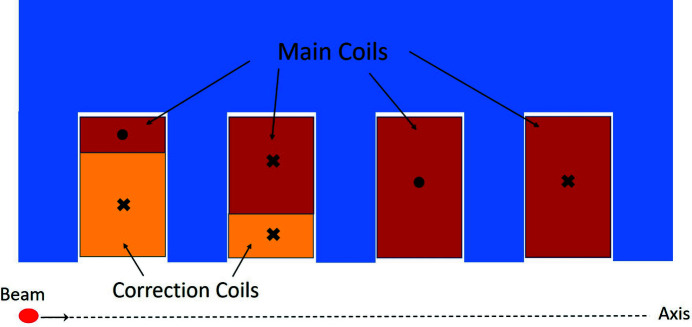
Winding configuration of the first four coils grooves on one core-end. The main coil packs (red rectangles) and the correction coil packs (yellow rectangles) are indicated in the figure. The directions of the coils are marked with dots and crosses.

In addition to the main coils, the SCU also has correction coils wound in the first and second grooves on each core end. The coil winding configuration for the last four packs on one core end is shown in Fig. 3 ▸. The red rectangles indicate the main coils, and the yellow ones indicate the correction coils. Each groove contains a full pack with 72 turns of coils. The first correction coil pack consists of 59 turns and the second one consists of 20 turns. The winding direction for each coil pack is marked as dots and crosses in the figure.

When a beam passes through the undulator along the axis, the beam trajectory is controlled by the magnetic field. For a vertical planar undulator, the longitudinal and horizontal component of the magnetic field on-axis is zero (*B*_*z*_ = 0, *B*_*x*_ = 0). The beam transverse motion depends on the vertical magnetic field *B*_*y*_ as 

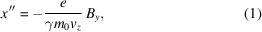

where the double prime indicates the derivative with respect to *z*, *v*_*z*_ is the beam velocity in the longitudinal direction, *e* is the beam charge, γ is the relativistic factor, and *m*_0_ is the electron rest mass. Assuming the initial angular deflections [*x*′(0), *y*′(0)] are zero, the horizontal trajectory angle along *z* is given by 

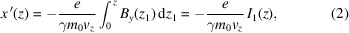

where *I*_1_(*z*) is defined as the first integral of the magnetic field. Then the electron beam transverse displacement can be obtained, 

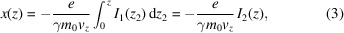

where *I*_2_(*z*) is the second integral of the magnetic field.

**Figure 4:**
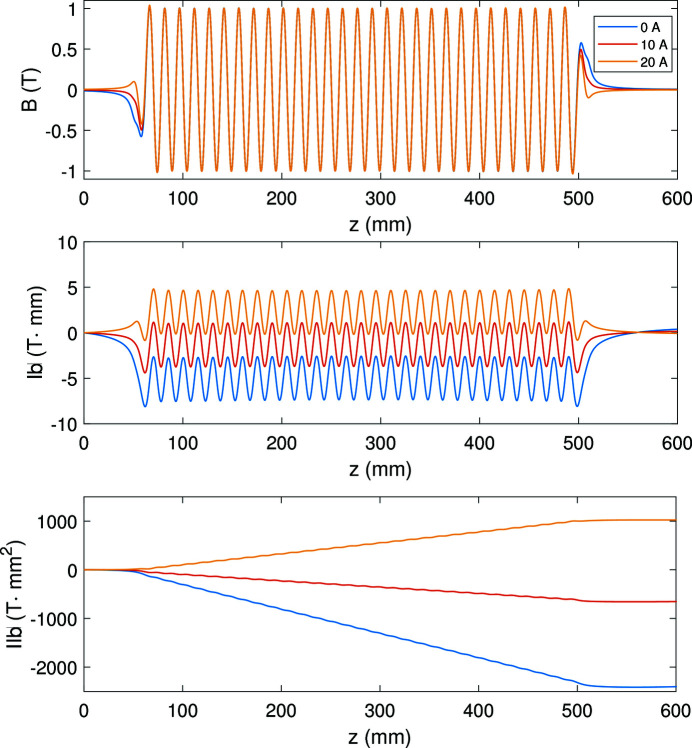
The magnetic field on-axis (top) and the corresponding first integrals (middle) and second integrals (bottom) with 0 A, 10 A and 20 A correction currents, respectively.

The main function of the correction current is to adjust the first and second integrals of the magnetic field on-axis which determine the beam trajectory along the undulator. With suitable correction current, the beam trajectory can be optimized. The top plot in Fig. 4 ▸ presents the magnetic fields on-axis with the same main current (434 A) while the correction currents are different. The corresponding first and second integrals of the magnetic fields are given in the middle and bottom plots, respectively. In this figure, the magnetic first and second integrals are improved with 10 A correction current.

## 3. SCU prototype

**Figure 5:**
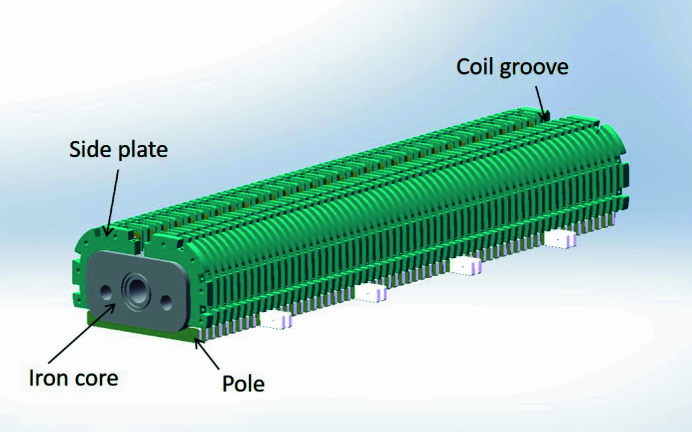
Structure of the SCU mock-up.

The precision of the manufacturing and fabrication of the SCU determines the magnetic field quality directly. The structure of the mock-up is shown in Fig. 5 ▸.

The core of the SCU is made of pure iron DT4. The poles are also made of pure iron and are inserted into the slots on the iron core. The precision of the pole length is about 10 µm. The side plates made of G10 constitute a series of grooves. These grooves are connected for winding the coils continuously. We choose round NbTi/Cu wires with a diameter of 0.6 mm to wind the coils. The coils are wound vertically in a continuous scheme with a single strand on each iron core. An NbTi/Cu wire is wound in one groove in one direction, and then jumps to the next groove wound in the opposite direction.

**Figure 6:**
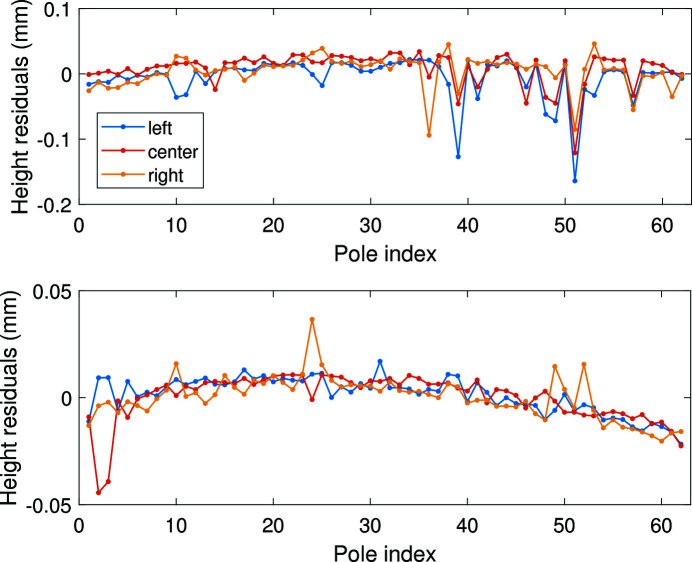
Residuals of the measured pole heights for one mock-up coil.

After assembling and winding, the mock-up coils were impregnated to reinforce the structure. In addition, the gap plane took a secondary processing to improve the uniformity of the pole height. We measured the pole height of the two mock-up coils. Each pole had three measurement points – on the left, the middle and the right. The residuals of the pole heights are given in Fig. 6 ▸. The RMS errors of the pole heights are 25 µm for the first mock-up and 9.8 µm for the second one. The first mock-up coil did not meet the tolerance requirement due to the poor manufacturing accuracy. The second mock-up was produced more precisely and the RMS error of the pole height was reduced significantly.

**Figure 7:**
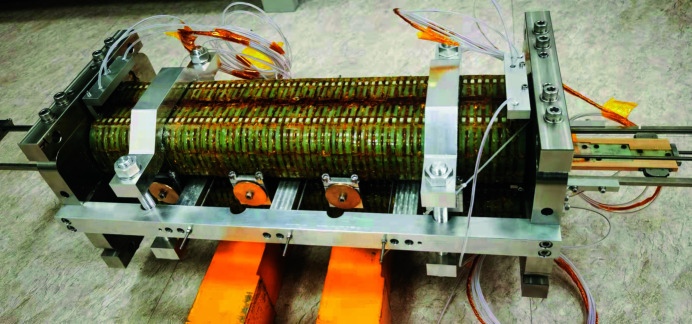
Photograph of the SCU prototype after fabrication.

The mock-up coils were mounted together using a frame to ensure a good alignment during the fabrication. The iron cores were clamped together on stainless steel blocks to maintain the magnetic gap during the measurement. The frame can also support and align the guide rail of the Hall probe sledge in the middle of the undulator gap. Fig. 7 ▸ shows a photograph of the SCU prototype after fabrication in the laboratory.

## 4. Vertical test system

**Figure 8:**
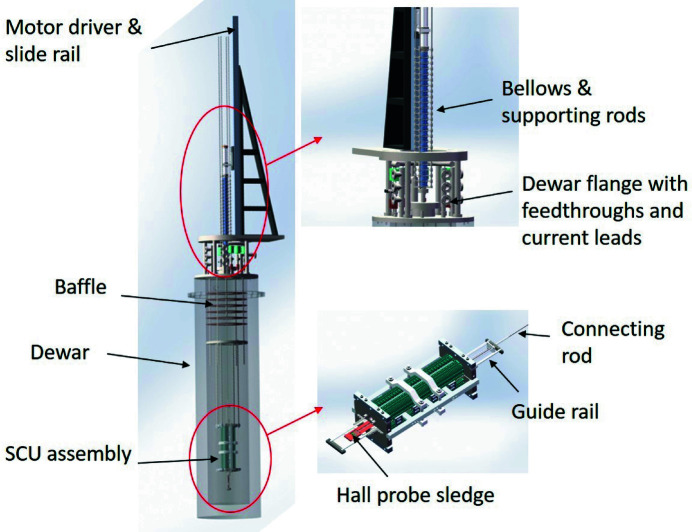
Schematic drawing of the vertical test system.

During the vertical test, the SCU is submerged in liquid helium in a Dewar to be trained at 4.2 K temperature to reach a maximum operation current. Fig. 8 ▸ shows the setup of the vertical test system.

The assembled SCU prototype was hung in a Dewar to be submerged in liquid helium. The SCU assembly was equipped with a Hall probe sledge guided by rails mounted in the middle of the gap. The sledge was connected to the top flange of the bellows by an aligned guide rod. A motor-driven linear slide rail was used to pull the motion structure including the bellows and the Hall probe sledge to measure the magnetic field on-axis. The bellows was specially designed to be supported by four external guide rods which stabilize the bellows during the measurement.

Three power supplies were used to power the SCU, one for the main coils and the other two for the correction coils. In order to ensure safety during the test, a specially designed quench protection system was used to protect the SCU from superconductivity loss by converting the magnetic stored energy to thermal energy as well as by cutting off the power supplies rapidly. The moving control and data acquisition program was developed based on *LabVIEW* software.

**Figure 9:**
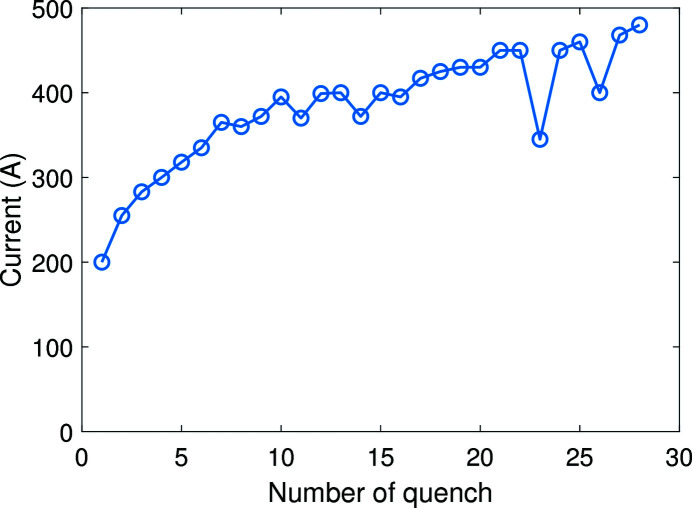
Graph of the SCU mock-up quench training.

The superconducting mock-up coils were trained in liquid helium. Fig. 9 ▸ gives the training curve of the SCU prototype. After 28 quenches, the maximum operation current reaches 480 A and the peak magnetic field on-axis reaches 1 T at 450 A.

## 5. Magnetic field analysis

**Figure 10:**
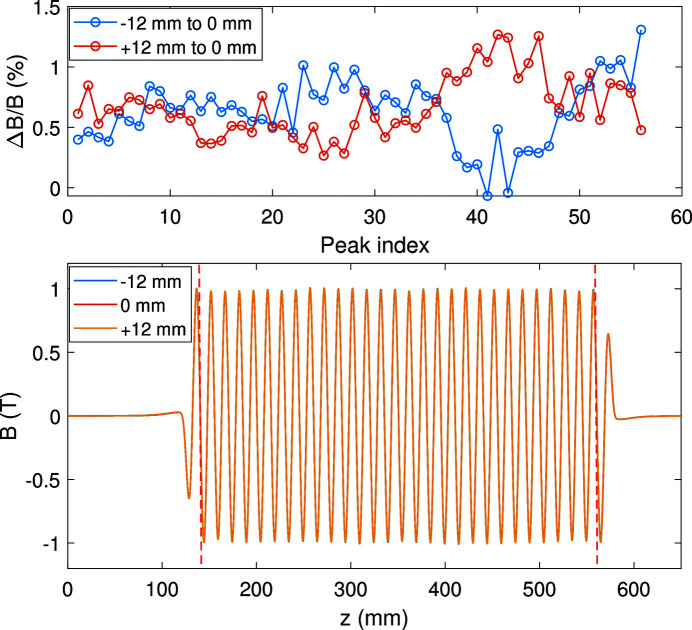
Local field map of the SCU measured by three Hall probes spaced 12 mm apart. The deviation of the peak values between the off-center probes and the central probe are given as a percentage.

As mentioned previously, three Hall probes were mounted on a sledge in the *x*-direction at 12 mm distance apart. The sledge can be pulled along the undulator guided by a stainless steel rail. The magnetic field distribution was measured in steps of 0.2 mm. Fig. 10 ▸ shows a field map of the magnetic field with 450 A main current and 25 A correction current. The peak field reaches 1 T. The middle part of the magnetic field is between the two red dashed lines. The deviations of the middle magnetic field peaks for the two off-centered Hall probes with respect to the central probe are calculated as a percentage.

The simulation results show that the magnetic field at *x* = ±12 mm decays by less than 0.1%. In our measurement, the off-center fields are both stronger than the center field. According to Grau *et al.* (2012 ▸), a roll angle of 1.3° would explain this effect. At the same time the three probes are not identical and the accuracy of the calibration plays an important role.

**Figure 11:**
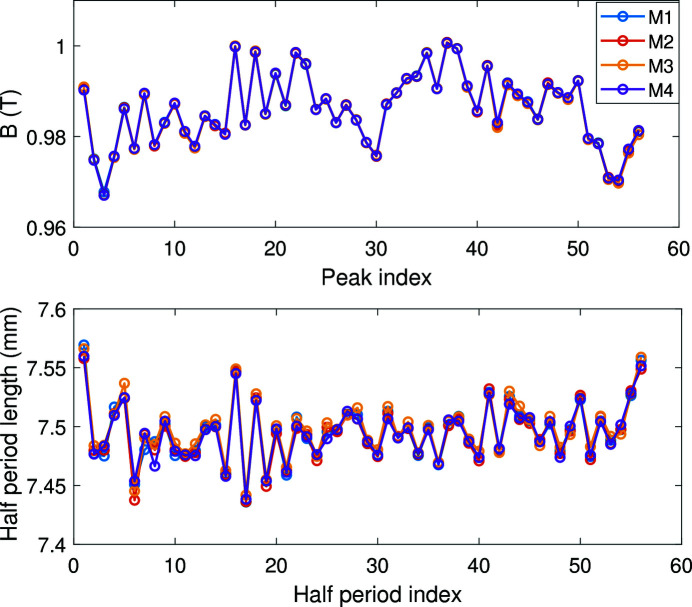
Absolute values of field peak and the half-period length for the center field with 450 A main current and 25 A correction current for four repeated measurements.

**Figure 12:**
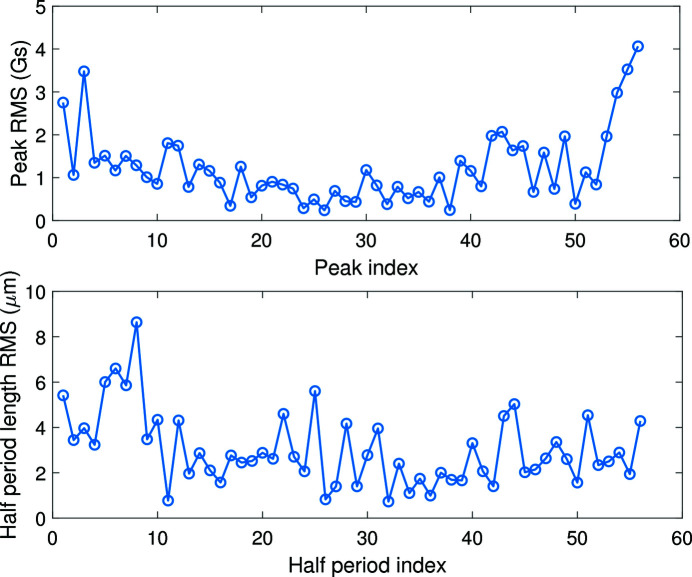
The measurement RMS error of the peak magnetic field and the half-period length.

The middle part of the magnetic field ideally has a sine shape. We repeated the measurement four times at 450 A main current and 25 A correction current. The absolute value of each field peak and the half-period length are evaluated as shown in Fig. 11 ▸. The measurement RMS errors for each peak and for each half-period length are given in Fig. 12 ▸. The RMS errors are under 5 G for the peak fields and are under 10 µm for the half-period lengths.

**Figure 13:**
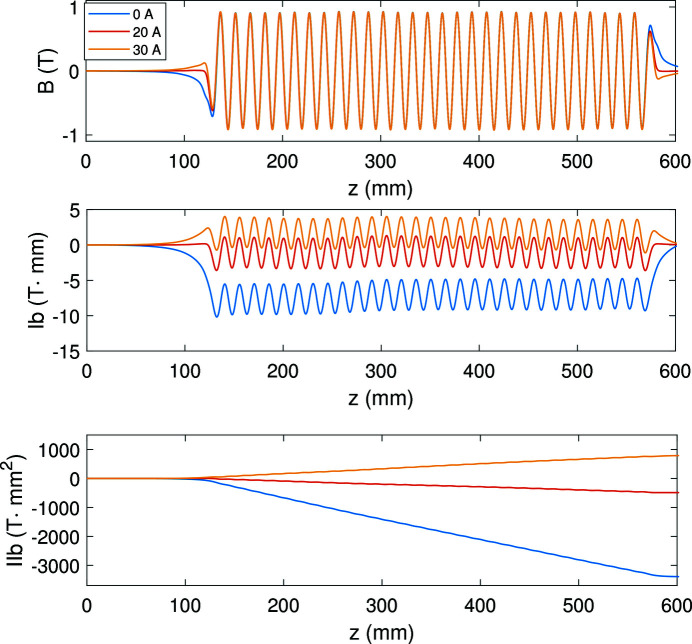
Hall probe measured magnetic field map (top) and the corresponding first field integrals (middle) and the second field integrals (bottom) with 400 A main current and 0 A, 20 A, 30 A correction currents.

As mentioned before, correction coils are used to adjust the first and second magnetic field (Doose *et al.*, 2011 ▸). During the measurement campaign, we studied the influence of the correction coils on the magnetic field with different currents. Fig. 13 ▸ shows the measured magnetic fields and the corresponding first and second integrals with 400 A main current and different correction currents.

The correction coils clearly optimized the first and second magnetic field integrals with 20 A current. The second integral was adjusted from −3400 T mm^2^ to −487 T mm^2^, which verified the correction ability of the correction coil winding configuration.

Another important parameter for assessing the magnetic field quality is the phase error. Electron beams are deflected due to the Lorenz force when passing through an undulator, and therefore continuously emit photons in a narrow cone around the forward direction. The optical phase of an undulator relies on the phase slip between the electron beam and the emitted photon. Equation (4) gives the phase difference between the emitted photon and the electron beam (Li *et al.*, 2011 ▸),

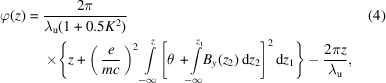

where λ_u_ is the undulator period length, *K* is the deflection parameter, *e* is the electron charge, *m* is the electron mass, and *c* is the light velocity. Here, θ is the so-called ‘launch angle’, defined to compensate the initial kick at the beginning of the periodic part of the magnetic field.

**Figure 14:**
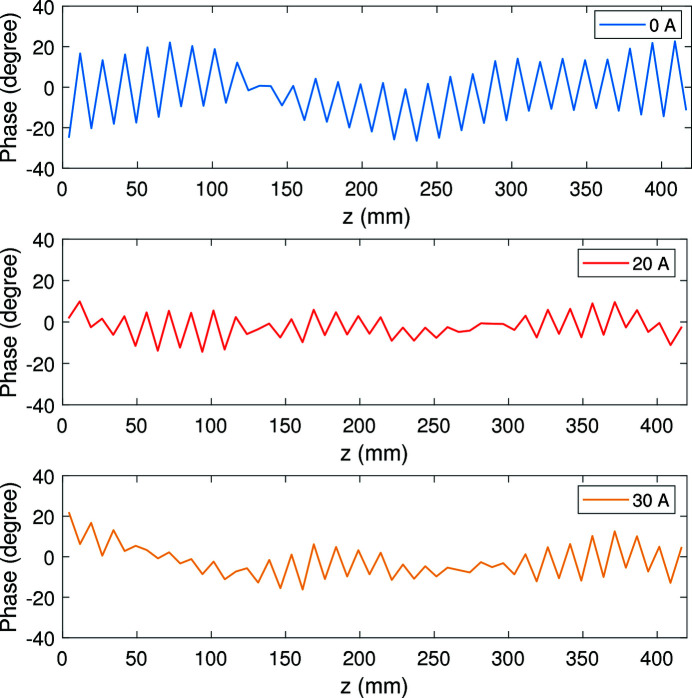
Phase maps with 400 main current and 0 A (top), 20 A (middle), 30 A (bottom) correction currents.

The maximum acceleration of the electron beam takes place at the pole center where the beam trajectory is parallel to the axis. Therefore the RMS phase error is calculated from the phase on each regular pole center. For the magnetic fields as given in Fig. 13 ▸, we calculated the corresponding phase maps as shown in Fig. 14 ▸. The phase RMS errors are 14.8°, 6.1° and 8.2° for 0 A, 20 A and 30 A correction currents, respectively.

**Figure 15:**
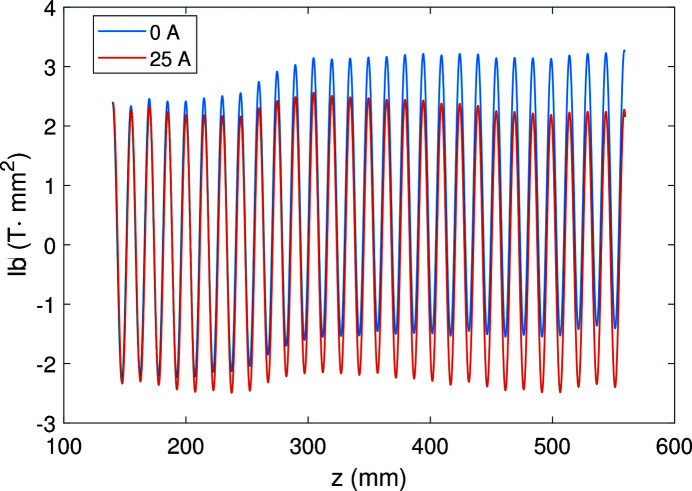
The first integral of the middle part of the magnetic field at 450 A main current with 0 A and 25 A correction current.

A suitable correction current can optimize the phase error of the magnetic field. According to equation (4), the first integral of the magnetic field determines the phase function. Here we compare the magnetic fields at 450 A main current while the correction currents are 0 A and 25 A. Fig. 15 ▸ shows the first integral of the middle part of the magnetic field. As we can see, the middle field first integral is straighter with the 25 A correction current, leading to a smaller phase error of about 6.9°. Meanwhile, the phase error for the 450 A main current without correction current is 16.3°.

**Figure 16:**
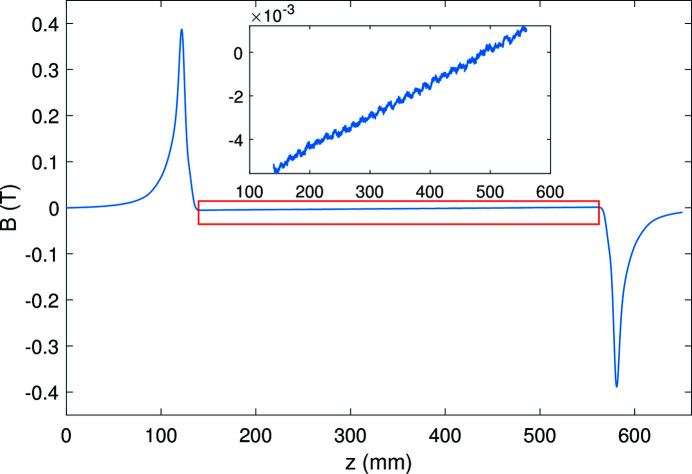
Magnetic field on-axis generated by correction coils with 20 A current.

For permanent-magnet undulators, the end magnets only correct the end fields and the phase error is optimized by shimming. For the SCU, our vertical test results show that the correction coils with a suitable current can not only adjust the end field to optimize the first and second field integrals but also affect the middle part of the magnetic field. We measured the magnetic field with 20 A current in the correction coils while the main current is zero. Fig. 16 ▸ shows the magnetic field distribution on-axis. The magnetic field generated by the correction coils mainly concentrates on the end parts of the SCU. However, the middle part of the correction field is not zero, and has a linear relationship with respect to the longitudinal distance.

For the main coils, the end coils can generate a magnetic field similar to the correction field along the undulator. The end-field part deteriorates the first and second field integrals, while the middle-field part disturbs the sinusoidal periodic field leading to a phase error increase. With suitable current, the correction-coil-generated magnetic field can counteract the field generated by the end coils of the main coils. Therefore, the correction coils have the ability to improve the first and second magnetic field integrals, as well as the phase error. As we can see in Figs. 13 ▸ and 14 ▸, the magnetic field integrals and phase error are both improved with the 20 A correction current. It is worth noting that the main purpose of the correction coils that we designed is to adjust the magnetic field integrals. The ability to improve the phase error is an additional benefit and needs further study in the future.

## 6. Conclusion

The ID group at IHEP recently developed its first prototype of a 0.5-m-long SCU. This prototype was designed using the simulation program *OPERA-3D* giving a very short period length of 15 mm and 7 mm gap. After manufacturing and fabrication, this prototype was cryogenic trained using a vertical test facility. During the test, the SCU was hung in a Dewar and submerged in liquid helium. Three Hall probes were mounted on a sledge driven by the motion system and guided by rails. The Hall probes measured the magnetic field on the gap center along the longitudinal axis.

After quench training, the maximum current applied in the main coils reached 480 A and the peak magnetic field exceeded 1 T. Regarding the quality of the measurement system, the RMS error of four repeated measurements was better than 5 G for the peak field and is under 10 µm for the half-period length. We also tested the correction coils with different currents. The measurement results revealed that, with suitable current in the correction coils, the first and second integrals of the magnetic field can be optimized. Moreover, the phase error can also be improved with a suitable correction current. When the main current is 400 A, the phase error was reduced to 6.1° by applying a 20 A correction current, while this value is 14.8° without correction current.

As the first SCU prototype developed at IHEP, we have learned lessons and accumulated experiences from the production process and the vertical test, which will benefit the 1.5 m-long SCU development in the future.

## Figures and Tables

**Table 1:** Parameters of the SCU simulation model

Parameter	Value
Period length (mm)	15
Period number	30.5
Pole length (mm)	3
Coil groove section (mm × mm)	4.5 × 6.5
Core width (mm)	90
Core height (mm)	47
Gap (mm)	7
Peak field (T)	1
